# iPSCs derived from insulin resistant offspring of type 2 diabetic patients show increased oxidative stress and lactate secretion

**DOI:** 10.1186/s13287-022-03123-4

**Published:** 2022-08-20

**Authors:** Bushra Memon, Ahmed K. Elsayed, Ilham Bettahi, Noor Suleiman, Ihab Younis, Eman Wehedy, Abdul Badi Abou-Samra, Essam M. Abdelalim

**Affiliations:** 1 College of Health and Life Sciences, Hamad Bin Khalifa University (HBKU), Qatar Foundation, Education City, Doha, Qatar; 2 Diabetes Research Center, Qatar Biomedical Research Institute (QBRI), Hamad Bin Khalifa University (HBKU), Qatar Foundation (QF), PO Box 34110, Doha, Qatar; 3 Qatar Metabolic Institute, Academic Health System, Hamad Medical Corporation, Doha, Qatar; 4 Translational Research Institute, Academic Health System, Hamad Medical Corporation, Doha, Qatar; 5 Biological Sciences Department, Carnegie Mellon Institute in Qatar, Qatar Foundation, Education City, Doha, Qatar

**Keywords:** Genetic predisposition, Diabetes, iPSCs, Insulin resistance, Oxidative stress

## Abstract

**Background:**

The genetic factors associated with insulin resistance (IR) are not well understood. Clinical studies on first-degree relatives of type 2 diabetic (T2D) patients, which have the highest genetic predisposition to T2D, have given insights into the role of IR in T2D pathogenesis. Induced pluripotent stem cells (iPSCs) are excellent tools for disease modeling as they can retain the genetic imprint of the disease. Therefore, in this study, we aimed to investigate the genetic perturbations associated with insulin resistance (IR) in the offspring of T2D parents using patient-specific iPSCs.

**Methods:**

We generated iPSCs from IR individuals (IR-iPSCs) that were offspring of T2D parents as well as from insulin-sensitive (IS-iPSCs) individuals. We then performed transcriptomics to identify key dysregulated gene networks in the IR-iPSCs in comparison to IS-iPSCs and functionally validated them.

**Results:**

Transcriptomics on IR-iPSCs revealed dysregulated gene networks and biological processes indicating that they carry the genetic defects associated with IR that may lead to T2D. The IR-iPSCs had increased lactate secretion and a higher phosphorylation of AKT upon stimulation with insulin. IR-iPSCs have increased cellular oxidative stress indicated by a high production of reactive oxygen species and higher susceptibility to H_2_O_2_ -induced apoptosis.

**Conclusions:**

IR-iPSCs generated from offspring of diabetic patients confirm that oxidative stress and increased lactate secretion, associated with IR, are inherited in this population, and may place them at a high risk of T2D. Overall, our IR-iPSC model can be employed for T2D modeling and drug screening studies that target genetic perturbations associated with IR in individuals with a high risk for T2D.

**Supplementary Information:**

The online version contains supplementary material available at 10.1186/s13287-022-03123-4.

## Background

Type 2 diabetes (T2D) is the most common form of diabetes representing ~ 90% of diabetic patients worldwide. Insulin resistance (IR) in insulin target tissues and pancreatic beta cell dysfunction, which leads to a relative deficiency in insulin secretion, are the main pathological features for the development of T2D [1]. Both genetic and environmental factors contribute to the development of IR in the insulin target tissues; however, the genetic factors associated with the development of IR and T2D remain largely unknown in humans due to lack of suitable models reflecting human physiology. For example, studies of glucose metabolism in T2D patients could be hindered due to the defects in insulin secretion and insulin signaling, which are associated with hyperglycemia, because hyperglycemic patients usually have IR independent of genetic defects [2]. Previous reports showed that IR is developed prior to determination of beta cell dysfunction [3] and is genetically inherited in the offspring of T2D diabetic patients as well as identical twins discordant for non-insulin dependent diabetes [3–5].

The first-degree relatives of T2D patients that have IR are at the highest risk of developing the disease [6, 7]. Since they are considered to carry this genetic imprint of IR and T2D, multiple comprehensive clinical and skeletal muscle biopsy studies have been conducted on them which postulated defective post-insulin receptor (INSR) signaling events and mitochondrial function as underlying defect causing IR and T2D at molecular level. For example, a decreased mitochondrial density was associated with a decreased expression of lipoprotein lipase in the skeletal muscle of the offspring of T2D parents [8]. Additionally, skeletal muscle of IR offspring also exhibits a reduced insulin-stimulated ATP synthesis and phosphate transport as well as increased phosphorylation of IRS-1 which may together affect insulin-mediated glucose disposal and fatty acid metabolism in these subjects [9–11]. However, the degree of genetic contribution to IR in these studies could not be determined due to factors such as group-to-group variations in subject selection criteria and different ethnicities, as well as interference by other existing factors like hyperglycemia, hyperinsulinemia, ectopic lipid accumulation, and pro-inflammatory factors; this makes it difficult to explain the early genetic events responsible for IR [8–10, 12].

The Induced pluripotent stem cell (iPSC) technology can generate cells genetically identical to IR individuals, which can help in distinguishing between genetic and acquired defects in insulin sensitivity [13, 14]. In our previous study, we demonstrated that the link between IR and psoriasis disease is genetic as IR is inherited in the keratinocytes derived from iPSCs of psoriatic patients. [15]. Other studies showed that iPSCs generated from patients carrying insulin receptor (INSR) mutations, which causes a rare form of syndromic IR or Donohue syndrome, are inadequately self-replicative and show disruption of insulin signaling events and gene expression [16]. In addition, these patient-derived iPSCs established a direct link between mitochondrial size, number and function with IR [17]. Furthermore, skeletal myoblasts generated from these iPSCs reflected the effect of inherent IR on insulin-stimulated biological pathways and signaling [12]. Also, a recent study generated iPSCs from IR individuals that were classified using the results for a steady state plasma glucose (SSPG)-derived from an insulin suppression test but with no fixed standard describing the family history of T2D in those subjects [18, 19]. On the other hand, studying IR offspring of T2D parents, individuals at the highest genetic risk of developing T2D, may facilitate our understanding of the specific genetic contribution of IR in the development of T2D more robustly. Therefore, in the current study, we have established iPSC lines from IR offspring of T2D parents (IR-iPSCs) and studied genetic defects in iPSCs prior to their differentiation into any lineages. Our results showed that these IR-iPSCs recapitulate defects such as cellular oxidative stress, increased lactate secretion and defects in insulin signaling that are associated with T2D.

## Methods

### Euglycemic hyperinsulinemic clamps

The study subjects were selected from among a cohort of 60 subjects previously described [20, 21]. The subjects were young adult, normoglycemic (normal fasting glucose and normal response to 75 g oral glucose tolerance test), healthy (no medication and no chronic diseases), and with body mass index below 28. All subjects had an euglycemic hyper-insulinemic clamps with infusion of insulin at a constant dose of 40 mU/min/m^2^ of body surface area for 120 min. At the same time 20% glucose is infused at a different vein to maintain blood glucose at normal level. Plasma glucose level was measured every 5 min and the glucose infusion was adjusted to maintain plasma glucose around 5 mmol/L. The amount of glucose infused per min per kg of body weight (M value) reflects the total body sensitivity to insulin. The subjects selected for the current study were the 3 most and 4 least insulin sensitive subjects (referred to as insulin sensitive or IS, and insulin resistant or IR) (Table 1). In this study, only the IR subjects selected for generation of iPSCs were offspring of patients with T2D. The study was approved by the ethical committee of Hamad Medical Corporation and Qatar Biomedical Research Institute and all subjects gave a written consent.

**Table 1:** Clinical demographics of subjects selected for iPSC generation in this study

Subject	Age	Gender	BMI	*M* value	Insulin sensitivity
IR-1	40	Male	24	5	Insulin resistant
IR-2	28	Male	22.5	5.25	Insulin resistant
IR-3	30	Male	28	3	Insulin resistant
IR-4	30	Male	19.4	2	Insulin resistant
IS-1	27	Male	26.3	18.5	Insulin sensitive
IS-2	31	Male	27.5	18	Insulin sensitive
IS-3	27	Male	23.4	17.75	Insulin sensitive

### PBMC extraction, culture, and reprogramming to iPSCs

Peripheral blood mononuclear cells (PBMCs) were extracted from the blood using standard protocol employing Ficoll-Paque Premium reagent (17-1440-02, GE Healthcare). PBMCs were cultured in StemPro-34 SFM media and following 4–6 days of expansion after their isolation, were reprogrammed using the CytoTune-iPS 2.0 Sendai virus (ThermoFisher Scientific) following the manufacturer’s instructions and as we previously reported [22–24]. The reprogrammed cells were maintained in suspension for the first two days and were plated onto 1:80 matrigel-coated dishes on the third day in StemPro media without cytokines. Media was first transitioned to a 1:1 ratio of StemPro and mTESR-1 medium (Stem Cell Technologies) and then switched to mTESR-1 medium. Media was then changed on alternate days until colonies began to grow. Following day 10, when colonies grew bigger, they were picked under a microscope and replated on to separate Matrigel-coated dishes. When the picked colonies expanded, they were passaged onto 6-well plates and further expanded for subsequent passages, characterization and freezing. At least 3 clonal lines were maintained, characterized, and stocked from each PBMC sample.

### iPSCs culture and maintenance

iPSCs were maintained in mTESR-1 medium and routinely passaged when the colonies reached a 70% confluency. For passaging, 0.5 mM EDTA (Gibco) was used to gently dissociate the colonies which were then replated on Matrigel (Corning) in mTESR-1 media.

### Spontaneous differentiation of iPSCs and scorecard analysis

Spontaneous differentiation was performed as previously described [15]. Briefly, iPSCs were made into single cells using TRYPLE and transferred to ultra-low attachment plates in 10% knock-out serum (KSR) containing knock-out DMEM media with supplementation of 10 µM rock inhibitor (Y-27632) (first 24 h) to form embryoid bodies (EBs). For immunostaining for different lineage markers, following 7 days of suspension, few EBs were then re-plated on Matrigel and continued to spontaneously differentiate. The rest of the EBs were then continued to differentiate for up to at least 14 days prior to RNA extraction for scorecard analysis. cDNA was synthesized from 1 ug of the extracted RNA and then used for scorecard analysis using the TaqMan^®^ hPSC Scorecard™ Kit 96w fast assays (A15876; Life Technologies) which was run on a QuantStudio7 Flex Real-Time PCR system (Applied Biosystems) according to manufacturer's instructions. The hPSC-ScoreCard-template-QuantStudio7-96-well template was used, and the data analysis was done through the cloud-based TaqMan hPSC Scorecard analysis software (www.lifetechnologies.com/scorecarddata).

### Karyotyping

When iPSCs reached 50–60% confluency, they were treated with KaryoMAX Colcemide solution in hPSC culture media for 2 h. Then, the treated iPSCs were trypsinized and washed and treated with a hypotonic solution containing 75 mM potassium chloride for 20 min at 37 °C. The cells were then fixed with solution containing methanol/glacial acetic acid (3:1) in a dropwise manner. The fixed cell suspension was then dropped onto slides and maintained at 90 °C for 60 min. These slides were then treated with Giemsa stain and 20 metaphases were analyzed.

### RNA extraction, cDNA synthesis, PCR, and qPCR

RNA was extracted from the iPSCs collected with trizol reagent using the Direct-zol RNA Extraction Kit (Zymo Research). cDNA was synthesized for 1 ug of RNA using the Superscript IV First Strand Synthesis System (ThermoFisher Scientific). For conventional RT-PCR, 2X PCR Master Mix (ThermoFisher Scientific) was used for amplification of the specified genes. For quantitative PCR, GoTaq qPCR Master Mix (Promega) was used, and SYBR-Green was quantified using Quant Studio 7 system (Applied Biosystems). The primer list is listed in Additional file 1: Table S1.

### Immunofluorescence for pluripotency and germ layer markers

Cells were washed with PBS and fixed with 4% paraformaldehyde for 20 min at room temperature with gentle shaking. Cells were washed with tris buffer saline supplemented with 0.5% tween (TBST) and then permeabilized with phosphate buffer saline supplemented with 0.5% triton X-100 (PBST) for 30 min on the shaker. Finally, cells were blocked with 6% BSA in PBST overnight at 4C. Cells were then stained with primary antibodies (Additional file 2: Table S2) diluted in 3%BSA in PBST overnight at 4C. Following day, the stained cells were washed with TBST thrice, 10 min each, then incubated with Alexa-fluor-488 and -647 conjugated secondary antibodies at 1:500 dilution for 1 h at room temperature. Cells were then washed with TBST and stained with DAPI before imaging with an inverted fluorescence microscope.

### Western blotting

iPSCs were washed twice with PBS and serum starved for 3 h in KREB’s buffer (129 mM NaCl, 5 mM NaHCO_3_, 4.8 mM KCl, 2.5 mM CaCl_2_, 1.2 mM MgSO_4_, 1.2 mM KH_2_PO_4_, 10 mM HEPES, 1 mM glucose and 0.5% BSA adjusted to pH = 7.4) at 37C. Starved iPSCs were then washed with PBS and clones were treated with KREB + 10 nM insulin, KREB + 100 nM insulin and KREB + 0 nM insulin in separate wells. The treatment with insulin was done for 10 min at 37 °C and then media was aspirated, and plates were placed on ice. 100 ul of RIPA buffer containing 1X protease inhibitor + 2X phosphatase inhibitor was added in each well and homogenized. Lysates were then collected and stored at -80C. Lysates were spun down at maximum speed (> 8000 rpm) for 15 min and supernatants separated. Lysates were quantified with BCA protein assay and 20 ug of protein was denatured with 4X laemmli buffer + beta-mercaptoethanol at 97C for 5 min. Denatured proteins were separated on 10% SDS-PAGE gels along with protein ladder, transferred on nitrocellulose membrane and blocked with 15% blocking buffer (0% fat, skimmed milk powder) in TBST. Primary antibodies (Additional file 2: Table S2) were added at a concentration of 1:5000 in 2% blocking buffer overnight at 4C on a shaker. HRP-conjugated secondary antibodies were used at 1:10,000 conc at room temperature for 1 h. Membranes were washed and developed with West Pico development reagent.

### RNA-Sequencing

RNA was extracted from two clones for each sample (considered as biological replicates) using Direct-zol RNA Extraction Kit (Zymo Research). One µg of total RNA was used for the preparation of library for RNA-Sequencing. mRNA was captured using NEBNext (Poly A) mRNA Magnetic Isolation Kit (NEB, E7490) according to manufacturer's instructions. Immediately, preparation of libraries was started using NEBNext Ultra Directional RNA Library Prep Kit (NEB, E7420L). The quality of the library was assessed using the bioanalyzer and then sequenced on an Illumina Hiseq 4000 system. Raw data for reads was then converted to fastq using Illumina BCL2Fastq Conversion Software v2.20 following basic trimming. The FASTQ files of the RNA-seq reads were aligned to the UCSC hg38 reference genome using the STAR aligner with default parameters. The BAM file generated using the mapped reads was then processed with to extract exon–exon junctions. Finally, differential gene expression analysis was performed using CuffDiff with default parameters.

For normalization purposes, Cuffdiff computed fragment per kilobase per million mapped reads (FPKM). For identifying differentially expressed genes (DEGs), we only considered genes with FPKM > 0.5 and *P* value < 0.05. For visualization purposes (Heatmaps), the replicates were combined. Gene ontology for biological processes was performed using DAVID online software (https://david.ncifcrf.gov/). Only genes with log2 fold change > 0.5 and *P* value < 0.05 were considered for analysis.

### Flow-cytometry for DCFH-DA and Annexin V quantification

iPSCs maintained in mTESR-Plus medium were trypsinized from 1 well of a 12-well plate and collected with PBS containing 5% knock-out serum. Trypsinized iPSCs were then washed with PBS and each clone was distributed in two wells of 96-well V-bottom plates. 10 uM of DCFH-DA dye and 1X buffer (ab113851, DCFH-DA Cellular ROS Detection Assay Kit, Abcam) was diluted in KREB’s buffer and  ul of this was added to one well of each clone in the 96-well plate while only 1X buffer in KREB’s was added to the other well. The cells were placed on a shaker in the 37 °C incubator for 30 min. At the end of the incubation, 1 ul of 7-AAD dye was added to 100 ul of cells for only the wells containing DCFH-DA dye. Stained cells were incubated at room temperature for 10 min on a shaker and then spun down at 2000 rpm for 5 min. Cells were then collected in 300 ul of PBS and run on BD Accuri C6 Flow cytometer and data was processed using FlowJo. For Annexin V experiment, iPSCs were treated with 0.5 mM H_2_O_2_ in mTRES-Plus media for 3 h and then trypsinized as described above. Treated and untreated cells were distributed in wells for unstained control, PI staining and Annexin V staining. Annexin V and PI were added to the designated wells for individual staining as per the manufacturer’s instructions (Abcam). Following 10 min of incubation, the cells were collected and run on BD Accuri C6 flow-cytometry machine in separate channels. Results were processed using FlowJo.

### L-lactate secretion assay

iPSCs were washed twice and KREB’s buffer (1 mM glucose) was added to one well of 12-w plate per clone. Following 15 min of incubation, the supernatant from each clone was collected and spun down a 10 kDa column to separate lactate dehydrogenase. The supernatant was collected and incubated with the lactate substrate using the L-Lactate Assay kit (Abcam, ab65330), following manufacturer’s instructions. Protein was then collected using RIPA from the colonies for normalization of total protein collected for l-lactate secreted per clone.

## Results

### Generation of iPSCs from IR offspring of T2D patients

**Fig. 1:**
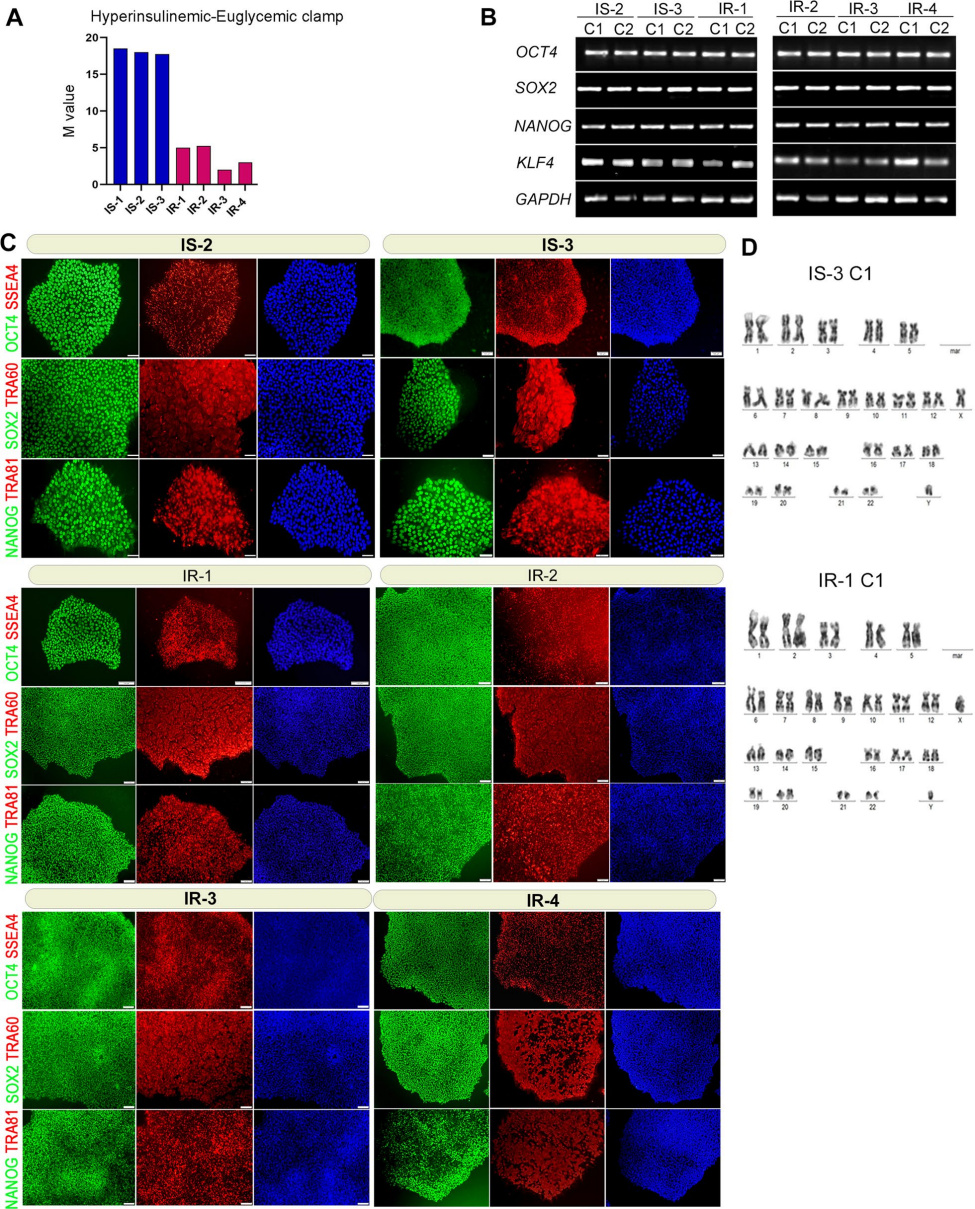
Insulin-resistant offspring of T2D patients and insulin-sensitive individuals generate pluripotent iPSCs. **A** M values indicating amount of glucose infused per min per kg of body weight as determined by hyperinsulinemic-euglycemic clamp for the recruited subjects. **B** Immunostaining for pluripotency markers for representative clones derived from each sample, **C** PCR for key pluripotency genes in IS and IR iPSCs, **D** karyotyping analysis of representative IS and IR iPSCs showing normal number of chromosomes. OCT4, green; SOX2, green; NANOG, green; TRA-60, red; TRA-81, red; SSEA-4, red. Scale bars = 100 µm

**Fig. 2:**
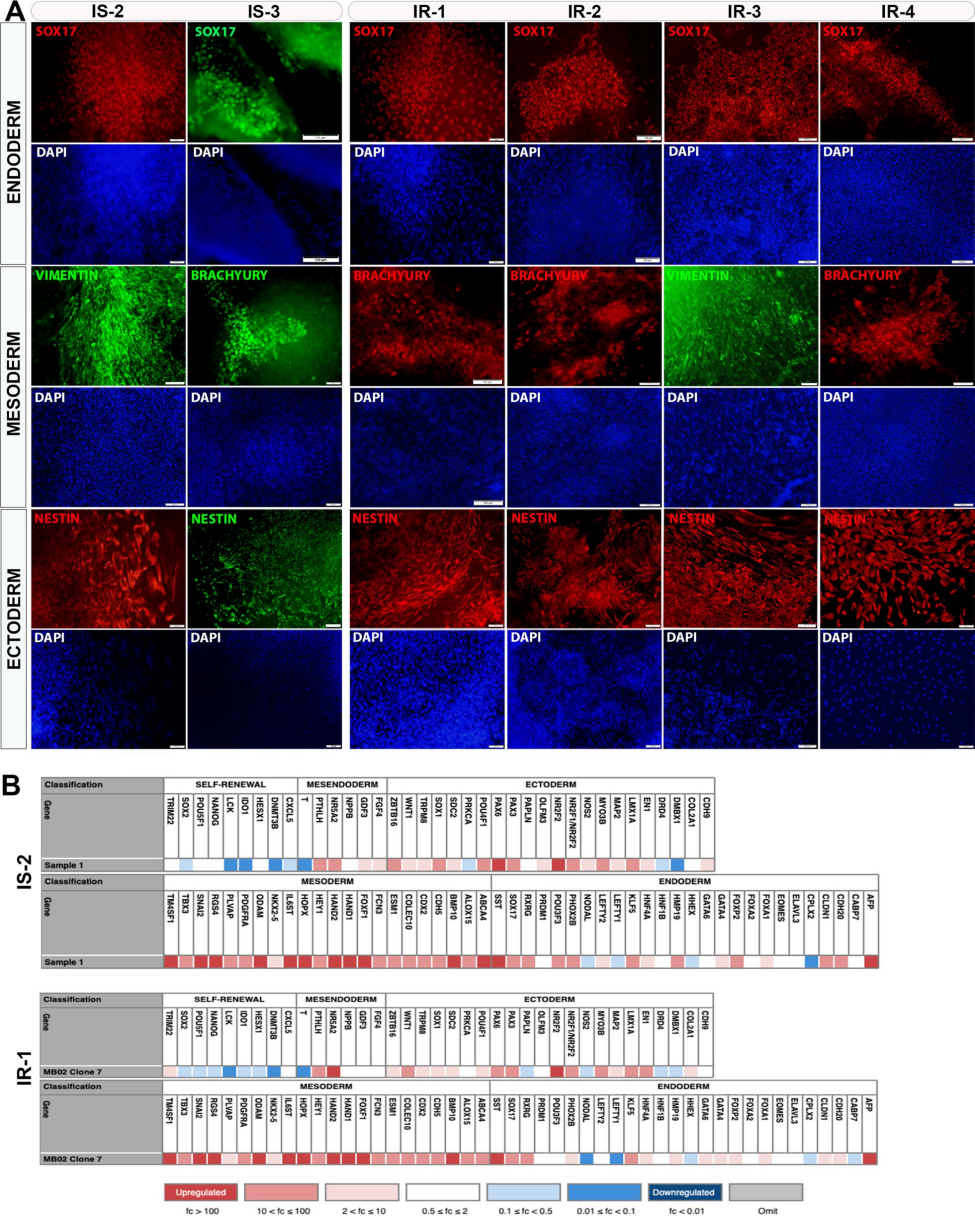
iPSCs derived from insulin-resistant offspring of T2D patients and insulin-sensitive individuals can differentiate to all germ layers. **A** Immunostaining analysis for the three germ layer markers; endoderm (SOX17), ectoderm (NESTIN) and mesoderm (BRACHYURY, VIMENTIN) in spontaneously differentiated embryoid bodies (EBs) from the generated iPSCs. **B** Scorecard analysis results for mRNA of representative IS and IR-iPSCs that were spontaneously differentiated as EBs showing downregulation of pluripotency markers and increased expression of the three lineage markers

The subjects who had the lowest M values (the amount of glucose infused per min per kg of body weight) in the euglycemic hyperinsulinemic clamp procedure were classified as insulin-resistant (IR) and those with highest M values were classified as insulin-sensitive (IS) (Table 1). iPSCs were generated from three IS control subjects, IS-1, IS-2, and IS-3 with M values 18.5, 18, and 17.75, respectively; and four IR subjects, IR-1, IR-2, IR-3, and IR-4 with M values 5, 5.25, 3, and 2, respectively (Fig. 1A). At least three iPSC lines were maintained for each subject and characterized extensively for pluripotency properties. The IS-iPSCs and IR-iPSCs showed adequate mRNA expression of key pluripotency transcription factors, including OCT4, SOX2, NANOG, and KLF4 (Fig. 1B) and loss of the exogenous Sendai virus backbone by RT-PCR (not shown). Furthermore, immunostaining analysis of the generated iPSC colonies revealed a high expression of pluripotency markers, OCT4, SOX2, NANOG, SSEA4, TRA-60, and TRA-81 (Fig. 1C). Our karyotype analysis confirmed the normal karyotypes of all generated iPSCs (Fig. 1D; Additional file 4: Fig. S1). Furthermore, to confirm the ability of the generated iPSCs to differentiate into cells from all three germ layers, we performed a spontaneous differentiation of the iPSCs by forming them into embryoid bodies (EBs) in suspension cultures. To perform immunostaining for germ layer markers, we plated the EBs on Matrigel following 7 days in suspension and continued their spontaneous differentiation until at least a total of 14 days. Immunostaining of replated EBs showed expression of key lineage-specific markers, including NESTIN (ectoderm), SOX17 (endoderm), and Vimentin and BRACHYURY (mesoderm) (Fig. 2A). Following spontaneous differentiation in suspension for a minimum of 14 days, we performed scorecard analysis from the extracted RNA of the EBs. The scorecard analysis quantified selected transcripts from each germ layer (endoderm, ectoderm, and mesoderm) and revealed that the pluripotency genes were significantly downregulated along with the upregulation of endodermal, ectodermal, and mesodermal genes in spontaneously differentiated EBs (Fig. 2B). This indicates that our iPSC lines are fully pluripotent.

### Transcriptome profiling of IR-iPSCs reveals dysregulated genes associated with insulin resistance

**Fig. 3:**
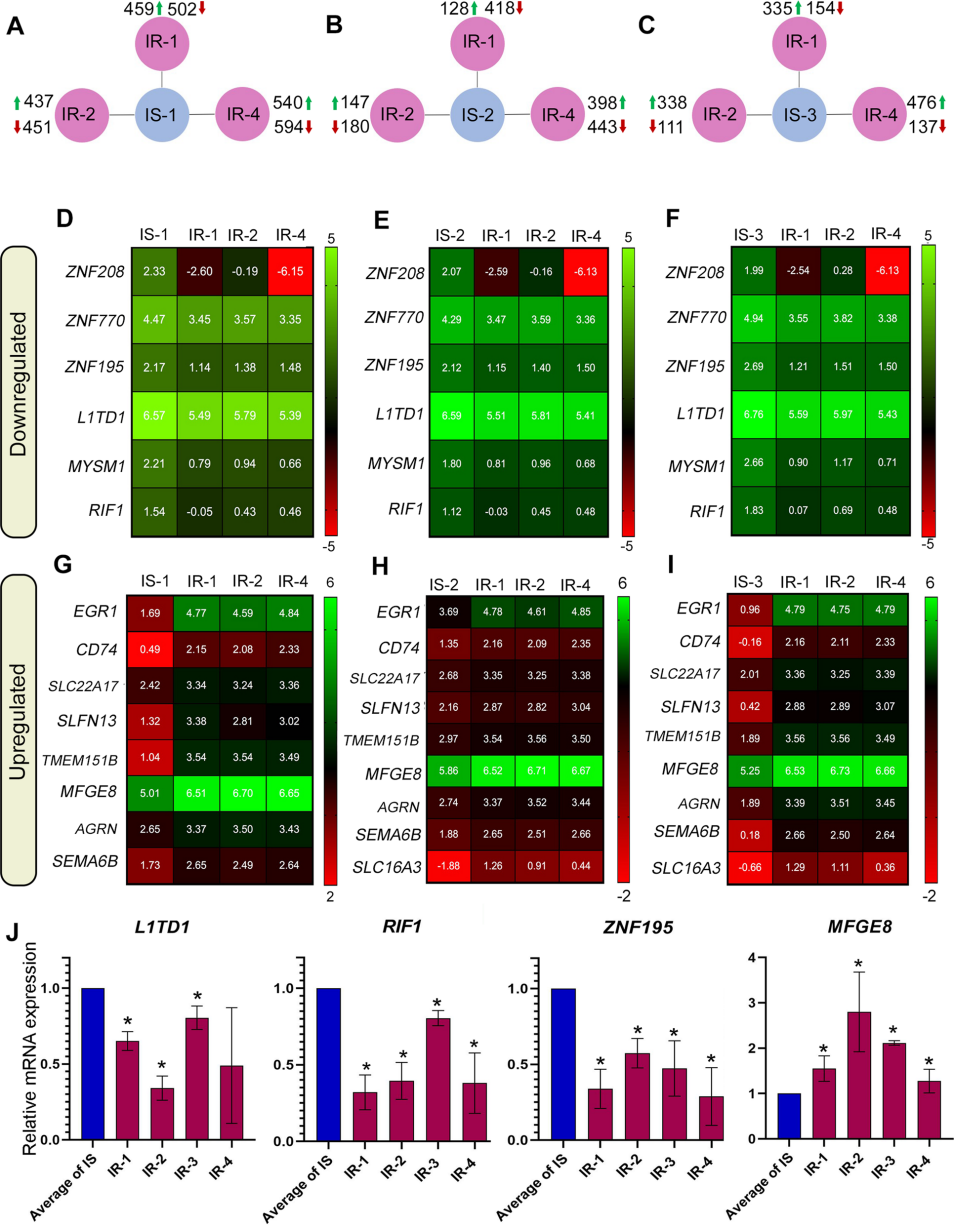
Transcriptomics of IS- and IR-iPSCs reveal differentially expressed genes (DEGs) in IR-iPSCs. **A**–**C** Summary of upregulated and downregulated DEGs for each comparison. Heat maps for **D**–**F** downregulated genes and **G**–**I** upregulated in all IR-iPSCs when compared individually to each of the IS-iPSCs based on RNA-Sequencing. Log2 of FPKM values of each IR-iPSC in comparison to IS-iPSC was plotted as heatmap. **J** Quantitative PCR results for select candidate genes identified from DEG analysis such as L1TD1, RIF1, ZNF195 and MFGE8 where delta CT for each IR-iPSC was normalized to the average delta CT for all IS-iPSC cell lines. (*n* = 2 clones), * *P* value < 0.05

To identify the complete spectrum of dysregulated mRNA expression in the IR-iPSCs, we performed RNA-Sequencing (RNA-seq) analysis for iPSCs generated from three IR and three IS individuals. We used two different iPSC lines derived from each sample and took their average to determine gene expression values. Each IR-iPSC sample (IR-1, IR-2, and IR-4) was compared with each IS-iPSC sample (IS-1, IS-2, and IS-3). The commonly dysregulated genes were then extracted following individual comparisons for each group. We found that 459, 437 and 540 genes were significantly upregulated in IR-1, IR-2, and IR-4 iPSCs when compared to IS-1 iPSCs, respectively (log2 fold change > 0.5, *P* value < 0.05) (Fig. 3A). In comparison to IS-2 iPSCs, 128, 147, and 398 genes were significantly upregulated in IR-1, IR-2 and IR-4 iPSCs, respectively (Fig. 3B). Additionally, 335, 338, and 476 genes were significantly upregulated in IR-1, IR-2, and IR-4 iPSCs, respectively, when compared to IS-3 iPSCs (Fig. 3C). Furthermore, we found that 502, 451, and 594 genes were significantly downregulated in IR-1, IR-2, and IR-4 iPSCs, respectively, compared to IS-1 iPSCs (Fig. 3A). A total of 418, 180, and 443 genes were downregulated IR-1, IR-2, and IR-4 iPSCs, respectively, when compared to IS-2 iPSCs (Fig. 3B). We found that 154, 111, and 137 genes were significantly downregulated in the IR-1, IR-2 and IR-4 iPSCs, respectively, in comparison to IS-3 iPSCs (log2 fold change > 0.5, *P* value < 0.05) (Fig. 3C).

The transcriptome analysis of the IR and IS iPSCs identified genes and pathways dysregulated in the IR individuals with parents having T2D. Assessment of differentially expressed genes (DEGs) highlighted 6 genes that were commonly downregulated in all IR-iPSCs when compared to each IS-iPSC group. Of these, we observed a downregulation of the zinc-finger family genes, including *ZNF208*, *ZNF770,* and *ZNF195 (*Fig. 3D–F). Other genes that were commonly downregulated include *L1TD1*, *MYSM1,* and *RIF1* (Fig. 3D–F). Genes commonly upregulated in the IR-iPSCs when compared to each IS-iPSC group include *EGR1* and *MFGE8,* which were previously associated with insulin resistance [25–30]*,* as well as *CD74, SEMA6B, SLFN13, TMEM151B, SLC22A17,* and *AGRN* (Fig. 3G–I). Our RT-qPCR analysis confirmed the significant upregulation of *MFGE8* and significant downregulation of the mRNA levels of *L1TD1*, *RIF1*, and *ZNF195* in all IR-iPSCs (IR-1, IR-2, IR-3, IR-4) compared to the average delta CT of the IS-iPSCs (IS-1, IS-2, IS-3) (Fig. 3J) (Additional file 3: Table S3).

### IR-iPSCs show increased cellular oxidative stress

**Fig. 4:**
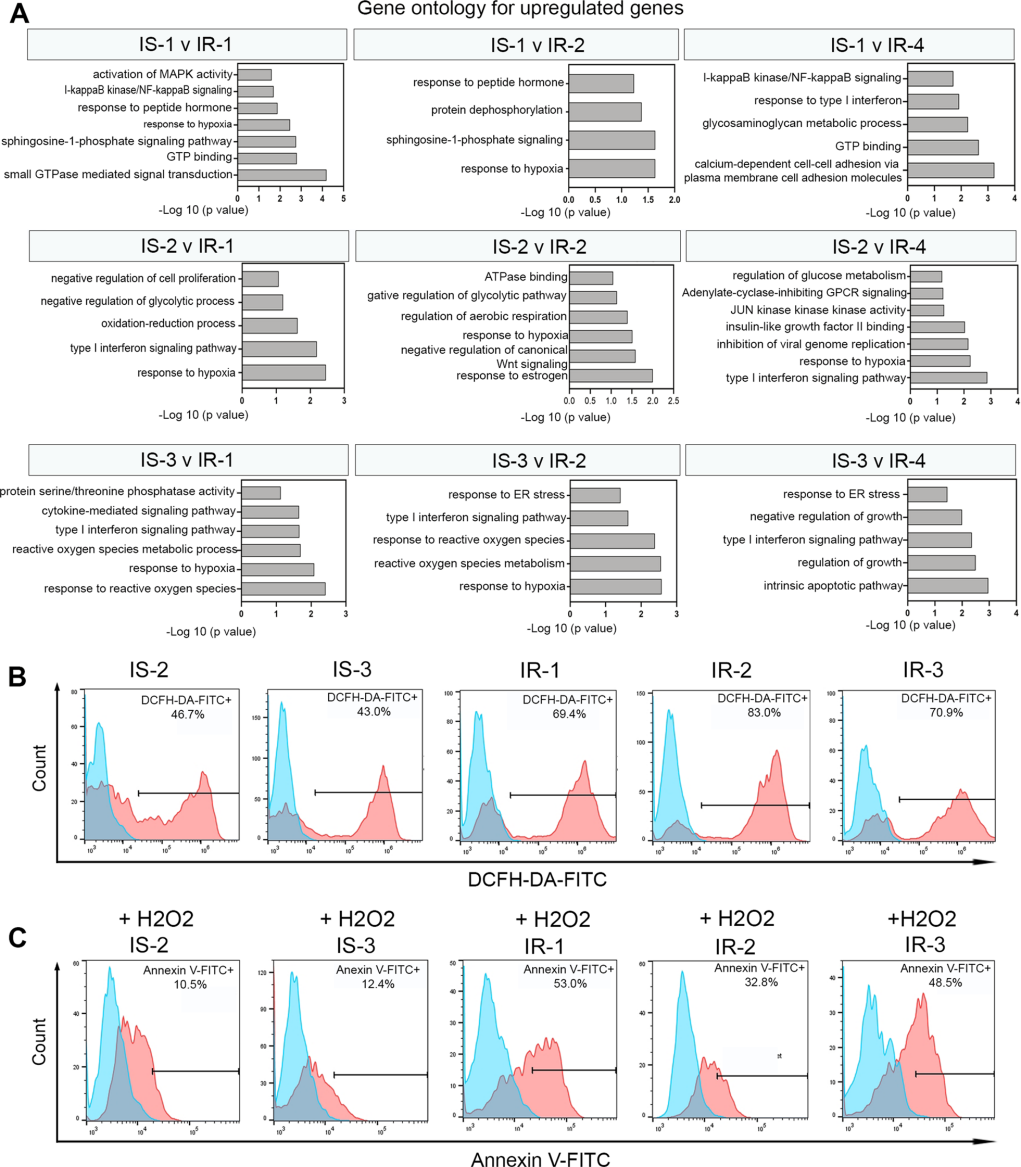
IR-iPSCs manifest increased cellular oxidative stress. **A** Gene ontology for upregulated genes in IR-iPSCs individually compared to IS-iPSCs obtained by RNA-Seq. **B** ROS quantification using the DCFH-DA by flow-cytometry in basal states showed increased expression in IR-iPSCs. **C** Annexin V quantification by flow-cytometry in iPSCs-treated with 0.5 mM H_2_O_2_ showed increased susceptibility of IR-iPSCs to oxidative stress

Gene ontology (GO) of the upregulated genes performed using DAVID software showed a significant enrichment of biological pathways associated with response to hypoxia and reactive oxygen species (ROS) in the IR-iPSCs. Specifically, biological processes such as “response to hypoxia”, “response to type I interferon”, “response to reactive oxygen species”, were upregulated in each of the IR-iPSCs when individually compared to each IS-iPSC sample (Fig. 4A). To confirm the elevated hypoxic stress in the IR-iPSCs, we performed a ROS quantification assay using the 2’-7’dichlorofluorescin diacetate dye (DCFH-DA) that, when oxidized by ROS, emits fluorescence. IR-iPSCs, under basal culture conditions, when treated with the DCFH-DA dye showed a higher fluorescence in the FL-1 channel compared to the IS-iPSCs. Thus, the results indicate that a higher percentage of ROS is observed in the IR-iPSCs (Fig. 4B, Additional file 5: Fig. S2A). Of note, in basal state, we observed a trend towards higher cell death in the IR-iPSCs compared to the IS-iPSCs as shown by the percentage of 7-AAD-positive iPSCs (Additional file 5: Fig. S2C), indicating that the IR-iPSCs may be more susceptible to cellular stress. Therefore, to validate if the IR-iPSCs are highly susceptible to oxidative stress, we treated the iPSCs with 0.5 mM of hydrogen peroxide (H_2_O_2_), a component of the cellular ROS, for 3 h to induce oxidative stress. We then quantified the levels of Annexin V using an apoptosis detection assay. Complementary to our results, we observed that upon treatment with 0.5 mM H_2_O_2_, the IR-iPSCs showed elevated level of Annexin V-FITC by flow-cytometry, indicating that the IR-iPSCs were more apoptotic and hence more susceptible to hypoxia-induced cellular stress when compared to the IS-iPSCs (Fig. 4C, Additional file 5: Fig. S2B).

### IR-iPSCs have increased lactate secretion

**Fig. 5:**
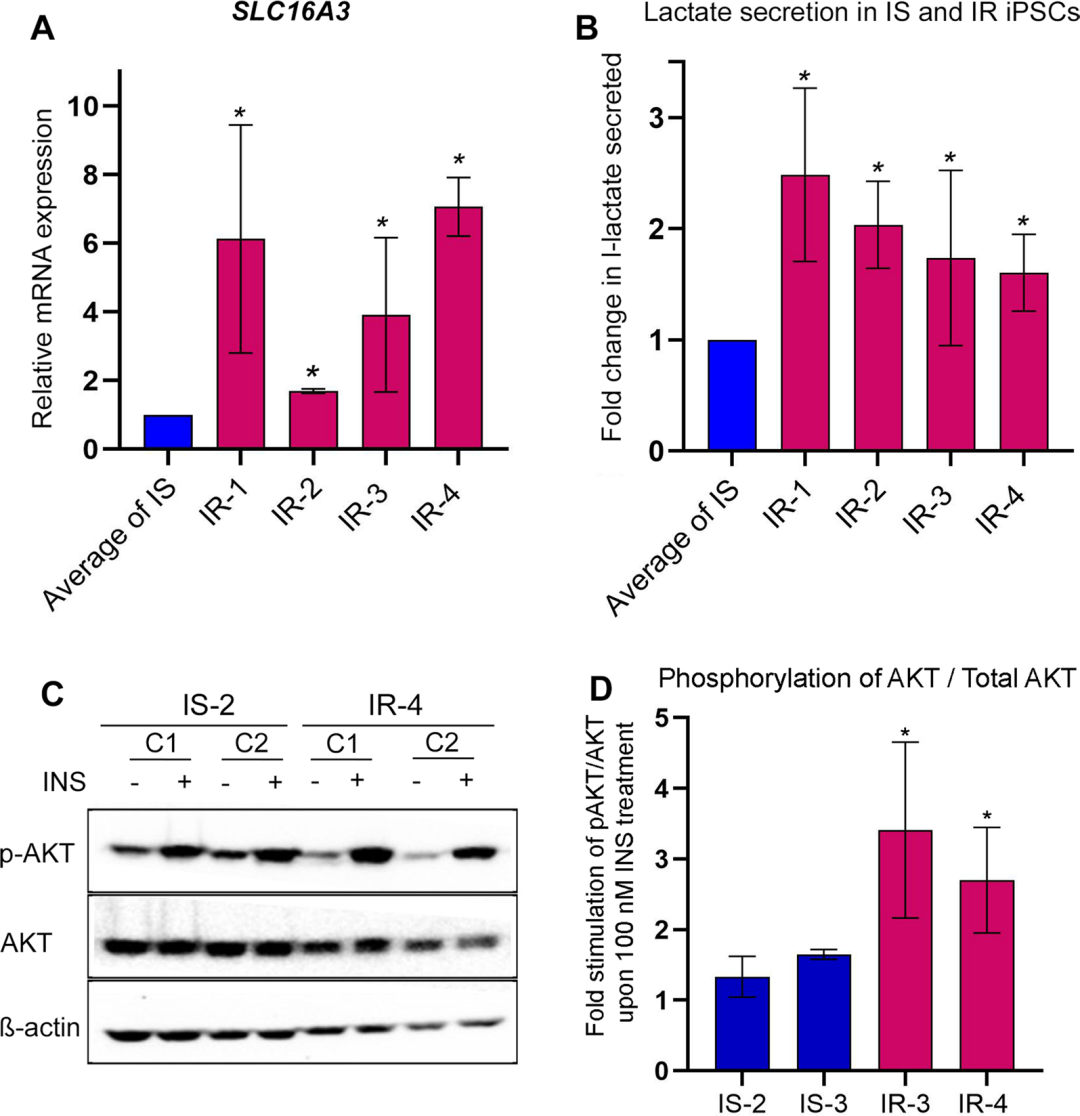
IR-iPSCs exhibit abnormal lactate secretion and are highly stimulated by insulin. **A** Quantitative PCR analysis for the lactate exporter gene *SLC16A3*. **B** Fold change in l-lactate secreted by IS- and IR-iPSCs in 15 min in KREB’s buffer. L-lactate secreted per ng of total protein for each IR-iPSC was normalized to average of that for IS-iPSCs in each experiment, **C**, **D** Western blot image for phosphorylation of AKT at Ser473 upon 0 nM or 100 nM insulin treatment in IS- and IR-iPSCs and its quantification performed using ImageJ software. *P value < 0.05, *n* = 2 clones

The RNA-seq results showed that the lactate exporter *SLC16A3* (also known as *MCT4*), one of the key genes regulated by hypoxia [31], was significantly upregulated in majority of the IR-iPSC versus IS-iPSC individual comparisons (Fig. 3G–I). We performed RT-qPCR to quantify the *SLC16A3* transcript and indeed observed a consistent upregulation of *SLC16A3* mRNA in the four IR-iPSC samples (IR-1, IR-2, IR-3, and IR-4) compared to the IS-iPSCs (Fig. 5A). To evaluate the functional relevance of the increased *SLC16A3* expression, we assessed lactate secretion under normal culture conditions across IS- and IR-iPSCs. Upon 15 min of serum starvation, the l-lactate secretion by IR-iPSCs measured using a lactate probe, normalized to their total protein content, was observed to be higher than the IS-iPSCs (Fig. 5B). However, this augmented levels of l-lactate secretion in IR-iPSCs may be unrelated to increased *SLC16A3* over-expression as it is unclear whether SLC16A3 is the major lactate exporter in iPSCs. On the contrary, the higher l-lactate secretion in IR-iPSCs is likely associated with their hypoxic state and is indicative of their increased anaerobic respiration process in basal state due to the prevalent oxidative stress.

### IR-iPSCs show increased phosphorylated AKT

The PI3K/AKT pathway plays a key role in regulating pluripotency and self-renewal of iPSCs [32–34]. Since PI3K/AKT pathway is also a major mediator of downstream events upon activation of insulin signaling, we sought to determine the effect of IR on phosphorylated AKT in our IR-iPSCs. We performed western blotting for lysates of IS- and IR-iPSCs that were treated with 100 nM insulin following serum starvation. Interestingly, we observed an increased stimulation capacity of the IR-iPSCs to insulin as revealed by increased phosphorylation of AKT at Serine 473 in the IR-iPSCs compared to IS-iPSCs (Fig. 5C, D). This increased phosphorylation of AKT in response to 100 nM insulin was consistently observed in multiple IR-iPSC lines.

## Discussion

Individuals at genetic risk for T2D tend to develop IR several years before developing glucose intolerance [35, 36]. The development of IR in insulin-target cells involves genetic and acquired factors. However, the genetic basis for the development of IR in those cells is not fully understood at molecular level. In this study, we generated a novel iPSC model for investigating genetic defects associated with IR in offspring of T2D parents, which are at a high risk for developing T2D. To our knowledge, this is the first study to utilize the iPSC approach for examining IR in this high T2D risk population. Our results showed that IR-iPSCs harbor dysregulated genes, which may play a role in T2D development.

Our results implicate the downregulation of ZNF family members such as ZNF208, ZNF195, and ZNF770 in the IR-iPSCs. ZNF family of transcription factors were known for their role in developmental processes [37]. The association of genetic polymorphism of *ZNF208* with coronary artery disease (CAD) was previously discovered in a Chinese population [38]. Herein, our data unveils the association of ZNF208, ZNF195, and ZNF770 with IR for the first time. Pluripotency-related genes, L1TD1 and RIF1, were also downregulated in the IR-iPSCs compared to IS-iPSCs. RIF-1 is a key DNA damage response regulator whereby it also maintains genome integrity. Depletion of RIF-1 from mouse embryonic stem cells (ESCs) results in loss of pluripotency and proliferation [39]. Interestingly, severe IR due to INSR mutations has been shown to negatively impact self-renewal of iPSCs [16]. Furthermore, we found that the key genes that may potentiate IR such as EGR1 and MFGE8 were significantly upregulated in the IR-iPSCs in comparison to IS-iPSCs. MFGE8 is an integrin ligand that promotes uptake of fatty acids leading to obesity [29]. A missense variant is MFGE8 is also found to be associated with T2D and cardiovascular disorders [30]. In addition, MFGE8 participates in auto-inhibition of the insulin receptor by cooperating with integrin αvβ5 in an insulin signaling feedback loop [28]. EGR1 has been also shown to participate in IR development by causing Serine phosphorylation of IRS-1 in adipocytes and loss of EGR1 in mice protected them from obesity, IR, hyperlipedimia, and hyperinsulinemia [25]. Therefore, overall, our transcriptome analysis of IR-iPSCs confirmed a genetic foundation for IR in the offspring of T2D patients.

Furthermore, our findings indicate an augmented response to hypoxia and oxidative stress that is inherent in the IR-iPSCs highlighting a limited oxygen availability in these cells. Transcriptome analysis highlighted an increased response to hypoxia in the IR-iPSCs, which was validated by our ROS quantification assay using DCFH-DA dye. Additionally, we showed that that the IR-associated genetic aberrations inherited in the IR-iPSCs make them more susceptible to H_2_O_2_-induced oxidative stress and apoptosis. Oxidative stress is one of the major players in the pathophysiology of IR [40–42]. Also, it has been suggested that the metabolic imbalances in the skeletal muscle of the first-degree relatives of diabetics causes oxidative stress that could aggravate IR [43]. Our RNA-seq results indicated a genetic inheritance of increased oxidative stress associated with IR in IR offspring of T2D parents. Furthermore, the IR-iPSCs exhibited an increased lactate secretion in untreated, basal conditions, indicative of the inherent hypoxia and may also suggest a resultant increased glycolysis or anaerobic respiration [44]. While increased lactate secretion may also correspond to the higher expression of the lactate transporter *SLC16A3* mRNA expression, the presence of HIF1α-response elements (HRE) in the *SLC16A3* regulatory region suggests that hypoxia or oxidative stress may have been the drivers of *SLC16A3* mRNA upregulation in the IR-iPSCs [31, 45]. Previous studies demonstrated that the lactate secretion is increased in hyperinsulinemic state, which is in line with our results, and an increased lactate level may lead to IR in the skeletal muscle [46, 47].

Complementing our results showing increased oxidative stress and lactate secretion by the IR-iPSCs, we found an increased phosphorylation of AKT upon stimulation with insulin in IR-iPSCs. AKT activation is a key mediator for the insulin signaling further downstream [48]. Studies have suggested a diminished stimulation of AKT2 and AKT3 at Ser473 site was observed upon activation with insulin in skeletal muscle of diabetic humans [49]. The skeletal muscle of IR offspring of T2D showed a 60% reduction in the phosphorylation at Ser473 of AKT [10]. However, in the current study, we observed the opposite effect on AKT activation in iPSCs derived from IR offspring of T2D parents. Furthermore, multiple studies have indicated a link between hypoxia, lactate, and increased AKT activation. Phosphorylation of AKT has been shown to be increased in hypoxic and stress conditions as well as under the influence of lactate [50, 51]. Therefore, it is likely that the increased activation of AKT in the IR-iPSCs upon insulin treatment in our study is due to their inherent hypoxia and increased lactate levels. Lactate has been previously shown to protect cancer cells from glucose starvation via the AKT/mTORC/BCL-2 axis [52]; therefore, the increased lactate and higher stimulation capacity of AKT may play protective roles in the IR-iPSCs.

Oxidative stress due to increased ROS suggests a compromised mitochondrial function; and the two phenomena lead to a vicious cycle, thus, aggravating IR. Indeed, mitochondrial dysfunction abolishes insulin sensitivity in insulin-target tissues and plays a major role in T2D pathogenesis [53, 54]. The impact of mitochondrial dysfunction on hindered hepatic or skeletal muscle activity in the IR milieu, shall be addressed in our future studies on insulin-target tissues derived from the IR-iPSCs.

## Conclusions

Although several genetic and environmental factors are known to be involved in the development of IR in individuals at risk for T2D, the exact signaling pathways underlying the development of genetic IR and its progression to T2D remain unknown. Since the environmental or acquired/epigenetic factors are lost while retaining only the genetic imprint of the individual in the generated iPSCs, our study focused on deciphering the inherited genetic defects that cause IR using iPSCs. While other studies focused on iPSCs from IR individuals in the common population regardless of their family history of T2D; we generated a novel human iPSC model for IR offspring of T2D patients, that are at a high risk of T2D, in order to gauge the molecular consequences of inherited IR more robustly in a specific cellular context. Using our model, we demonstrated a genetic association for novel genes with IR, that previously did not have a function related to IR. We also established a genetic basis for increased hypoxia and oxidative stress in IR individuals, as well as for elevated levels of l-lactate secretion by these individuals. Our future studies will focus on dissecting the molecular perturbances present in hepatocytes and skeletal muscle derived from IR-iPSC model, as well as other insulin-target tissues, for studying their development and functional performance. While in this study we investigated iPSCs derived from only 4 IR offspring that were unrelated; our results highlighting the existence of common genetic defects in these IR-iPSCs support our study to be a stepping-stone towards understanding the molecular outline for how the genetic defects lead to IR development, specifically in the IR offspring of T2D patients. Indeed, our results pave the way for developing extensive libraries for IR-iPSCs derived from high T2D risk groups, that could further identify therapeutic targets that are inherited in these groups.

## Supplementary Information

**Additional file 1: Table S1.** List of primers used for PCR and qPCR.

**Additional file 2: Table S2.** List of antibodies used for immunostaining, flow cytometry and Western blotting.

**Additional file 3: Table S3.** FPKM values for select genes for IR samples compared to IS samples derived from RNA-Seq.

**Additional file 4: Fig. S1.** Karyotyping analysis of different clones used in this study that were generated from IS and IR samples.

**Additional file 5: Fig. S2.** **A** Quantification of DCFH-DA+ iPSCs under normal culture conditions, **B** Annexin V+ iPSCs upon treatment of 0.5 mM H_2_O_2_, and **C** 7-AAD+ iPSCs under normal cell culture conditions across different IS and IR cell lines. *p value < 0.05, *n* = 2 clones.

## Data Availability

The data of this study are available from the corresponding author upon reasonable request.

## Abbreviations

ESCs: Embryonic stem cells
INSR: Insulin receptor
iPSCs: Induced pluripotent stem cells
IR: Insulin resistance
IS: Insulin sensitive
T2D: Type 2 diabetes
