# Supplementary material for: iPSCs derived from insulin resistant offspring of type 2 diabetic patients show increased oxidative stress and lactate secretion
Source: Stem Cell Res Ther. 2022 Aug 20;13:428. doi: 10.1186/s13287-022-03123-4 (PMC9392338; doi:10.1186/s13287-022-03123-4)
Supplement: Supplementary file 1 — Additional file 1: Table S1. List of primers used for PCR and qPCR. [file 13287_2022_3123_MOESM1_ESM.docx]

**Supplementary Table 1: List of primers used for PCR and qPCR**

| **Target gene** | **Forward / reverse sequence 5’-3’** |
| --- | --- |
| OCT4 | GACAGGGGGAGGGGAGGAGCTAGG/ CTTCCCTCCAACCAGTTGCCCCA AAC |
| SOX2 | GGGAAATGGGAGGGGTGCAAA AGAGG/ TTGCGTGAGTGTGGATGGGATTG GTG |
| KLF4 | CCCAA TTACCCATCCTTCCT/  ACGATCGTCTTCCCCTCTTT |
| NANOG | CATGA GTGTGGATCCAGCTTG/  CCTGAATAAGCAGATCCATGG |
| MFGE8 | TGGCCAGTCATGAGTACCTG/  AGCTCGTGGGGTACAATCTC |
| L1TD1 | TCCCACAAAAGGAAGAAATAAATC/ GCTCTATGCTTTGAGTCTATTAGGG |
| ZNF195 | GTTGACGTTCAGGGATGTGG/  AGACAGTGAGACCAACGGAG |
| RIF1 | GGCCACAATCCCATCCATTC/  GAAGGGCTGCTGATTAACGG |
| GAPDH | ACGACCACTTTGTCAAGCTCATTTC/ GCAGTGAGGGTCTCTCTCTTCCTCT |
