# Supplementary material for: iPSCs derived from insulin resistant offspring of type 2 diabetic patients show increased oxidative stress and lactate secretion
Source: Stem Cell Res Ther. 2022 Aug 20;13:428. doi: 10.1186/s13287-022-03123-4 (PMC9392338; doi:10.1186/s13287-022-03123-4)
Supplement: Supplementary file 2 — Additional file 2: Table S2. List of antibodies used for immunostaining, flow cytometry and Western blotting. [file 13287_2022_3123_MOESM2_ESM.docx]

**Supplementary Table 2: List of antibodies used for immunostaining, flow cytometry and Western blotting**

| **Target** | **Company/Catalog** | **Dilution used** |
| --- | --- | --- |
| OCT4 | Cell Signaling Technology Cat# 9656 | 1:500 |
| SOX2 | Cell Signaling Technology Cat# 9656 | 1:500 |
| KLF4 | Cell Signaling Technology Cat# 9656 | 1:500 |
| NANOG | Cell Signaling Technology Cat# 9656 | 1:500 |
| TRA-60 | Cell Signaling Technology Cat# 9656 | 1:500 |
| TRA-81 | Cell Signaling Technology Cat# 9656 | 1:500 |
| SSEA4 | Cell Signaling Technology Cat# 9656 | 1:500 |
| SOX17 | OriGene Cat# TA500096 | 1:2000 |
| VIMENTIN | Cell Signaling Technology Cat# 3932 | 1:2000 |
| BRACHYURY | Abcam Cat# 140661 | 1:1000 |
| NESTIN | R and D Systems Cat# MAB1259 | 1:1000 |
| Phospho AKT (Ser473) | Cell Signaling Technology Cat# 4060 | 1:5000 |
| Pan AKT | Cell Signaling Technology Cat# 4691 | 1:5000 |
