# Supplementary material for: iPSCs derived from insulin resistant offspring of type 2 diabetic patients show increased oxidative stress and lactate secretion
Source: Stem Cell Res Ther. 2022 Aug 20;13:428. doi: 10.1186/s13287-022-03123-4 (PMC9392338; doi:10.1186/s13287-022-03123-4)
Supplement: Supplementary file 3 — Additional file 3: Table S3. FPKM values for select genes for IR samples compared to IS samples derived from RNA-Seq. [file 13287_2022_3123_MOESM3_ESM.docx]

**Supplementary Table 3: FPKM values for select genes for IR samples compared to IS samples derived from RNA-Seq**

**FPKM values for select genes for IR-1 compared to IS-1 derived from RNA-Seq.**

| **Gene** | **Gene name** | **Log2 fold change** | **P value** |
| --- | --- | --- | --- |
| ZNF208 | Zinc Finger Protein 208 | **-4.93091** | 5.00E-05 |
| RIF1 | Replication Timing Regulatory Factor 1 | **-1.59413** | 5.00E-05 |
| MYSM1 | Myb Like, SWIRM And MPN Domains 1 | **-1.42832** | 0.0003 |
| L1TD1 | LINE1 Type Transposase Domain Containing 1 | **-1.08914** | 0.00115 |
| ZNF195 | Zinc Finger Protein 195 | **-1.04406** | 0.00635 |
| ZNF770 | Zinc Finger Protein 770 | **-1.03329** | 0.005 |
| AGRN | Agrin Precursor | **0.714221** | 0.01665 |
| SEMA6B | Semaphorin 6B | **0.920241** | 0.0105 |
| SLC22A17 | **Solute carrier family 22 member 17** | **0.920255** | 0.0179 |
| MFGE8 | Milk fat globule-EGF factor 8 | **1.50132** | 5.00E-05 |
| CD74 | Cluster of Differentiation 74 | **1.66482** | 0.0007 |
| SLFN13 | Schlafen family member 13 | **2.21641** | 5.00E-05 |
| TMEM151B | transmembrane protein 151B | **2.49541** | 5.00E-05 |
| EGR1 | Early growth response protein 1 | **3.07928** | 5.00E-05 |

**FPKM values for select genes for IR-2 compared to IS-1 derived from RNA-Seq.**

| **Gene** | **Gene name** | **Log2 fold change** | **P value** |
| --- | --- | --- | --- |
| ZNF208 | Zinc Finger Protein 208 | **-2.50432** | 5.00E-05 |
| MYSM1 | Myb Like, SWIRM And MPN Domains 1 | **-1.26178** | 0.0004 |
| RIF1 | Replication Timing Regulatory Factor 1 | **-1.09234** | 0.00165 |
| ZNF770 | Zinc Finger Protein 770 | **-0.885838** | 0.00695 |
| ZNF195 | Zinc Finger Protein 195 | **-0.777705** | 0.01975 |
| L1TD1 | LINE1 Type Transposase Domain Containing 1 | **-0.768211** | 0.0123 |
| SEMA6B | Semaphorin 6B | **0.780251** | 0.0226 |
| SLC22A17 | **Solute carrier family 22 member 17** | **0.834578** | 0.021 |
| AGRN | Agrin Precursor | **0.864345** | 0.0049 |
| CD74 | Cluster of Differentiation 74 | **1.60747** | 0.0002 |
| MFGE8 | Milk fat globule-EGF factor 8 | **1.70581** | 5.00E-05 |
| SLFN13 | Schlafen family member 13 | **2.17914** | 5.00E-05 |
| TMEM151B | transmembrane protein 151B | **2.5163** | 5.00E-05 |
| EGR1 | Early growth response protein 1 | **2.9101** | 5.00E-05 |

**FPKM values for select genes for IR-3 compared to IS-1 derived from RNA-Seq.**

| **Gene** | **Gene name** | **Log2 fold change** | **P value** |
| --- | --- | --- | --- |
| ZNF208 | Zinc Finger Protein 208 | **-8.48121** | 0.0001 |
| MYSM1 | Myb Like, SWIRM And MPN Domains 1 | **-1.5619** | 5.00E-05 |
| L1TD1 | LINE1 Type Transposase Domain Containing 1 | **-1.18471** | 0.0001 |
| ZNF770 | Zinc Finger Protein 770 | **-1.13501** | 0.00025 |
| RIF1 | Replication Timing Regulatory Factor 1 | **-1.08287** | 0.0003 |
| ZNF195 | Zinc Finger Protein 195 | **-0.701554** | 0.02245 |
| AGRN | Agrin Precursor | **0.766044** | 0.00815 |
| SEMA6B | Semaphorin 6B | **0.909385** | 0.0043 |
| SLC22A17 | **Solute carrier family 22 member 17** | **0.936456** | 0.00715 |
| MFGE8 | Milk fat globule-EGF factor 8 | **1.63426** | 5.00E-05 |
| CD74 | Cluster of Differentiation 74 | **1.84065** | 5.00E-05 |
| SLFN13 | Schlafen family member 13 | **2.3707** | 5.00E-05 |
| TMEM151B | transmembrane protein 151B | **2.43751** | 5.00E-05 |
| EGR1 | Early growth response protein 1 | **3.13841** | 5.00E-05 |

**FPKM values for select genes for IR-1 compared to IS-2 derived from RNA-Seq.**

| **Gene** | **Gene name** | **Log2 fold change** | **P value** |
| --- | --- | --- | --- |
| ZNF208 | Zinc Finger Protein 208 | **-4.67024** | 5.00E-05 |
| RIF1 | Replication Timing Regulatory Factor 1 | **-1.16297** | 5.00E-05 |
| L1TD1 | LINE1 Type Transposase Domain Containing 1 | **-1.08921** | 5.00E-05 |
| MYSM1 | Myb Like, SWIRM And MPN Domains 1 | **-1.00367** | 0.00055 |
| ZNF195 | Zinc Finger Protein 195 | **-0.985375** | 0.0013 |
| ZNF770 | Zinc Finger Protein 770 | **-0.835072** | 0.0022 |
| TMEM151B | transmembrane protein 151B | **0.557651** | 0.04035 |
| AGRN | Agrin Precursor | **0.617023** | 0.0042 |
| MFGE8 | Milk fat globule-EGF factor 8 | **0.646393** | 0.00075 |
| SLC22A17 | **Solute carrier family 22 member 17** | **0.648623** | 0.03325 |
| SLFN13 | Schlafen family member 13 | **0.697406** | 0.01125 |
| SEMA6B | Semaphorin 6B | **0.760211** | 0.006 |
| CD74 | Cluster of Differentiation 74 | **0.79145** | 0.0168 |
| EGR1 | Early growth response protein 1 | **1.07123** | 0.00045 |
| SLC16A3 | Solute carrier family 16 member 3 | **3.12891** | 5.00E-05 |

**FPKM values for select genes for IR-2 compared to IS-2 derived from RNA-Seq.**

| **Gene** | **Gene name** | **Log2 fold change** | **P value** |
| --- | --- | --- | --- |
| ZNF208 | Zinc Finger Protein 208 | **-2.22023** | 5.00E-05 |
| MYSM1 | Myb Like, SWIRM And MPN Domains 1 | **-0.829092** | 0.00095 |
| L1TD1 | LINE1 Type Transposase Domain Containing 1 | **-0.763386** | 0.00025 |
| ZNF195 | Zinc Finger Protein 195 | **-0.709566** | 0.00845 |
| ZNF770 | Zinc Finger Protein 770 | **-0.680037** | 0.00625 |
| RIF1 | Replication Timing Regulatory Factor 1 | **-0.658018** | 0.00775 |
| SLC22A17 | **Solute carrier family 22 member 17** | **0.584584** | 0.032 |
| TMEM151B | transmembrane protein 151B | **0.598466** | 0.0199 |
| SEMA6B | Semaphorin 6B | **0.643523** | 0.0133 |
| SLFN13 | Schlafen family member 13 | **0.681361** | 0.00875 |
| CD74 | Cluster of Differentiation 74 | **0.75906** | 0.00865 |
| AGRN | Agrin Precursor | **0.787575** | 0.00035 |
| MFGE8 | Milk fat globule-EGF factor 8 | **0.872504** | 0.0001 |
| EGR1 | Early growth response protein 1 | **0.929614** | 0.0006 |
| SLC16A3 | Solute carrier family 16 member 3 | **2.80349** | 5.00E-05 |

**FPKM values for select genes for IR-3 compared to IS-2 derived from RNA-Seq.**

| **Gene** | **Gene name** | **Log2 fold change** | **P value** |
| --- | --- | --- | --- |
| ZNF208 | Zinc Finger Protein 208 | **-8.20396** | 5.00E-05 |
| L1TD1 | LINE1 Type Transposase Domain Containing 1 | **-1.17482** | 5.00E-05 |
| MYSM1 | Myb Like, SWIRM And MPN Domains 1 | **-1.12539** | 5.00E-05 |
| ZNF770 | Zinc Finger Protein 770 | **-0.926515** | 5.00E-05 |
| RIF1 | Replication Timing Regulatory Factor 1 | **-0.64682** | 0.00325 |
| ZNF195 | Zinc Finger Protein 195 | **-0.627666** | 0.0064 |
| TMEM151B | transmembrane protein 151B | **0.528343** | 0.0175 |
| SLC22A17 | **Solute carrier family 22 member 17** | **0.696148** | 0.00385 |
| AGRN | Agrin Precursor | **0.698753** | 0.0007 |
| SEMA6B | Semaphorin 6B | **0.780908** | 0.0008 |
| MFGE8 | Milk fat globule-EGF factor 8 | **0.809154** | 0.0003 |
| SLFN13 | Schlafen family member 13 | **0.881804** | 0.0003 |
| CD74 | Cluster of Differentiation 74 | **0.999785** | 5.00E-05 |
| EGR1 | Early growth response protein 1 | **1.15894** | 5.00E-05 |
| SLC16A3 | Solute carrier family 16 member 3 | **2.31819** | 5.00E-05 |

**FPKM values for select genes for IR-1 compared to IS-3 derived from RNA-Seq.**

| **Gene** | **Gene name** | **Log2 fold change** | **P value** |
| --- | --- | --- | --- |
| ZNF208 | Zinc Finger Protein 208 | **-4.5285959** | 5.00E-05 |
| MYSM1 | Myb Like, SWIRM And MPN Domains 1 | **-1.7614245** | 0.0028 |
| RIF1 | Replication Timing Regulatory Factor 1 | **-1.7539055** | 0.0025 |
| ZNF195 | Zinc Finger Protein 195 | **-1.4778651** | 0.0164 |
| ZNF770 | Zinc Finger Protein 770 | **-1.3812506** | 0.0139 |
| L1TD1 | LINE1 Type Transposase Domain Containing 1 | **-1.1590195** | 0.03165 |
| MFGE8 | Milk fat globule-EGF factor 8 | **1.290016377** | 0.01925 |
| SLC22A17 | **Solute carrier family 22 member 17** | **1.355410337** | 0.04575 |
| AGRN | Agrin Precursor | **1.499750622** | 0.0079 |
| TMEM151B | transmembrane protein 151B | **1.675726853** | 0.01485 |
| SLC16A3 | Solute carrier family 16 member 3 | **1.950540747** | 0.02185 |
| CD74 | Cluster of Differentiation 74 | **2.324875544** | 0.0106 |
| SLFN13 | Schlafen family member 13 | **2.465385655** | 0.00065 |
| SEMA6B | Semaphorin 6B | **2.48336555** | 0.00115 |
| EGR1 | Early growth response protein 1 | **3.836618774** | 5.00E-05 |

**FPKM values for select genes for IR-2 compared to IS-3 derived from RNA-Seq.**

| **Gene** | **Gene name** | **Log2 fold change** | **P value** |
| --- | --- | --- | --- |
| ZNF208 | Zinc Finger Protein 208 | **-1.7041273** | 0.0022 |
| MYSM1 | Myb Like, SWIRM And MPN Domains 1 | **-1.477953297** | 0.005 |
| ZNF195 | Zinc Finger Protein 195 | **-1.1674396** | 0.02645 |
| RIF1 | Replication Timing Regulatory Factor 1 | **-1.1285026** | 0.027 |
| ZNF770 | Zinc Finger Protein 770 | **-1.107846945** | 0.0282 |
| L1TD1 | LINE1 Type Transposase Domain Containing 1 | **-0.774964166** | 0.14675 |
| SLC22A17 | **Solute carrier family 22 member 17** | **1.248743569** | 0.0408 |
| MFGE8 | Milk fat globule-EGF factor 8 | **1.498946075** | 0.00715 |
| AGRN | Agrin Precursor | **1.633406651** | 0.00185 |
| TMEM151B | transmembrane protein 151B | **1.681169709** | 0.0042 |
| SLC16A3 | Solute carrier family 16 member 3 | **1.774703675** | 0.044 |
| CD74 | Cluster of Differentiation 74 | **2.278208167** | 0.0059 |
| SEMA6B | Semaphorin 6B | **2.333599966** | 0.00065 |
| SLFN13 | Schlafen family member 13 | **2.482815979** | 0.00105 |
| EGR1 | Early growth response protein 1 | **3.800003195** | 0.00075 |

**FPKM values for select genes for IR-3 compared to IS-3 derived from RNA-Seq.**

| **Gene** | **Gene name** | **Log2 fold change** | **P value** |
| --- | --- | --- | --- |
| ZNF208 | Zinc Finger Protein 208 | **-8.135533616** | 0.0066 |
| MYSM1 | Myb Like, SWIRM And MPN Domains 1 | **-1.968339342** | 0.0003 |
| ZNF770 | Zinc Finger Protein 770 | **-1.573977613** | 0.00315 |
| RIF1 | Replication Timing Regulatory Factor 1 | **-1.360953025** | 0.0142 |
| L1TD1 | LINE1 Type Transposase Domain Containing 1 | **-1.341795676** | 0.0101 |
| ZNF195 | Zinc Finger Protein 195 | **-1.211445136** | 0.03065 |
| SLC16A3 | Solute carrier family 16 member 3 | **0.992802871** | 0.26205 |
| SlC22A17 | **Solute carrier family 22 member 17** | **1.362426134** | 0.0358 |
| MFGE8 | Milk fat globule-EGF factor 8 | **1.397379749** | 0.0077 |
| AGRN | Agrin Precursor | **1.537444927** | 0.00525 |
| TMEM151B | transmembrane protein 151B | **1.586787415** | 0.0101 |
| SEMA6B | Semaphorin 6B | **2.443329736** | 0.00055 |
| CD74 | Cluster of Differentiation 74 | **2.474302216** | 0.0043 |
| SLFN13 | Schlafen family member 13 | **2.633071124** | 0.0001 |
| EGR1 | Early growth response protein 1 | **3.81731747** | 0.0002 |
