# Supplementary figures and images for: iPSCs derived from insulin resistant offspring of type 2 diabetic patients show increased oxidative stress and lactate secretion
Source: Stem Cell Res Ther. 2022 Aug 20;13:428. doi: 10.1186/s13287-022-03123-4 (PMC9392338; doi:10.1186/s13287-022-03123-4)

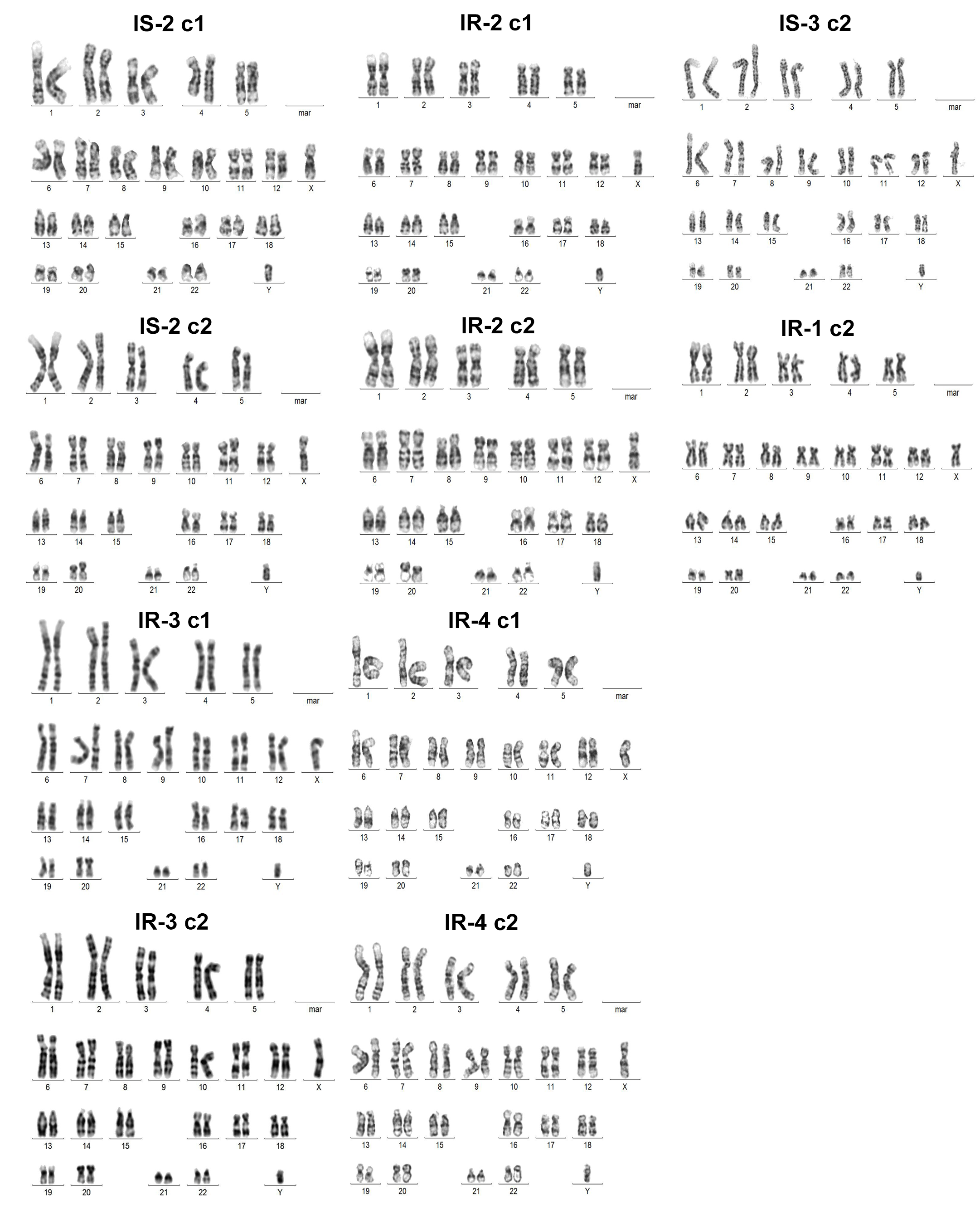

Supplement: Supplementary file 4 — Additional file 4: Fig. S1. Karyotyping analysis of different clones used in this study that were generated from IS and IR samples. [file 13287_2022_3123_MOESM4_ESM.tif]

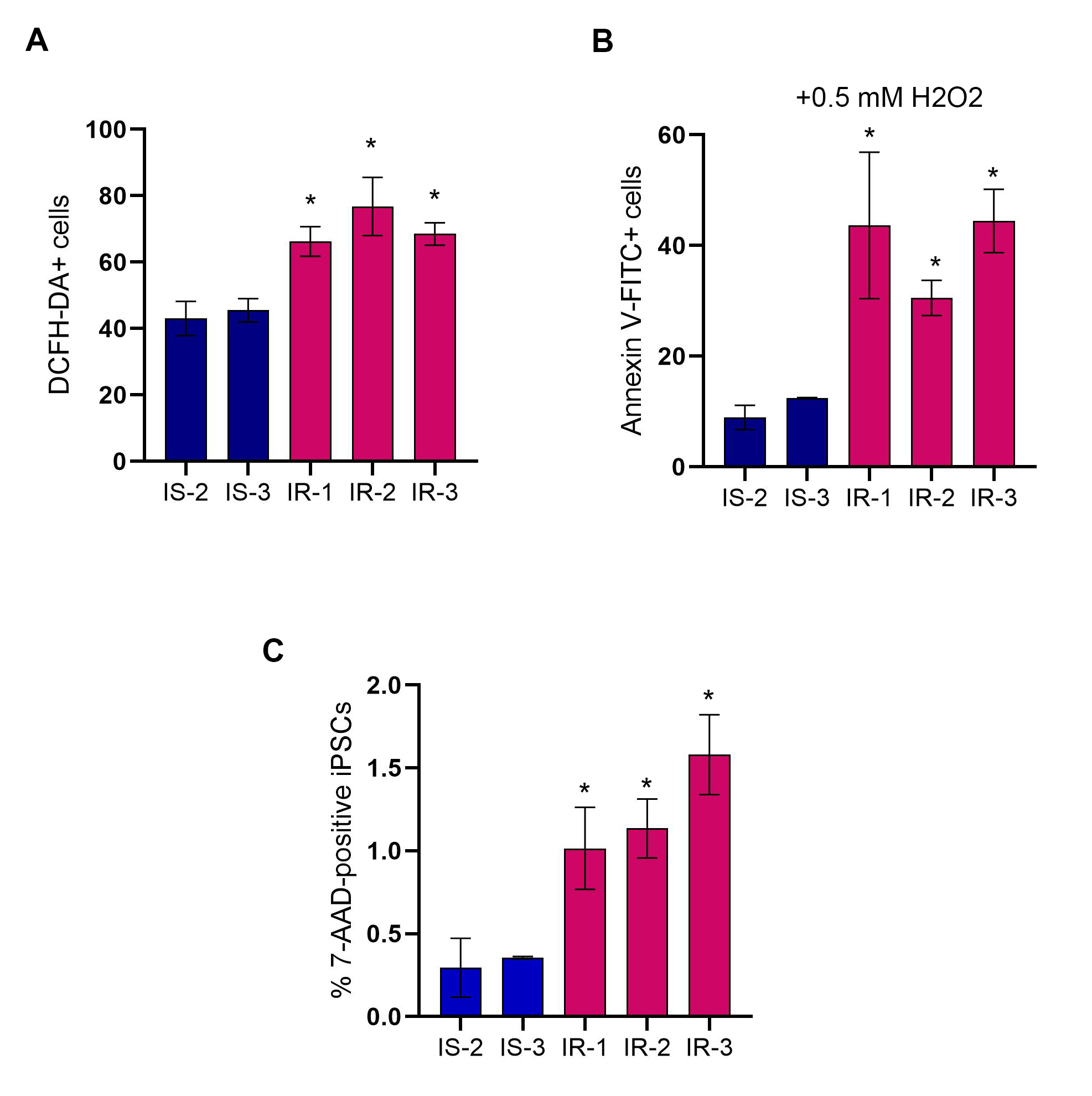

Supplement: Supplementary file 5 — Additional file 5: Fig. S2. A Quantification of DCFH-DA+ iPSCs under normal culture conditions, B Annexin V+ iPSCs upon treatment of 0.5 mM H2O2, and C 7-AAD+ iPSCs under normal cell culture conditions across different IS and IR cell lines. *p value < 0.05, n = 2 clones. [file 13287_2022_3123_MOESM5_ESM.tif]
